# A randomized trial of the efficacy and safety of quilizumab in adults with inadequately controlled allergic asthma

**DOI:** 10.1186/s12931-016-0347-2

**Published:** 2016-03-18

**Authors:** Jeffrey M. Harris, Romeo Maciuca, Mary S. Bradley, Christopher R. Cabanski, Heleen Scheerens, Jeremy Lim, Fang Cai, Mona Kishnani, X. Charlene Liao, Divya Samineni, Rui Zhu, Colette Cochran, Weily Soong, Joseph D. Diaz, Patrick Perin, Miguel Tsukayama, Dimo Dimov, Ioana Agache, Steven G. Kelsen

**Affiliations:** Genentech, Inc, 1 DNA Way, South San Francisco, CA 94080-4990 USA; Alabama Allergy & Asthma Center, Birmingham, AL USA; Allergy and Asthma Research Center PA, San Antonio, TX USA; Allergy Partners of New Jersey, Teaneck, NJ USA; Clínica Ricardo Palma, Lima, Peru; Trakia University, Stara Zagora, Bulgaria; Transylvania University, Faculty of Medicine, Brasov, Romania; Temple University School of Medicine, Philadelphia, PA USA

**Keywords:** Allergic asthma, Biomarkers, COSTA, IgE, M1 prime, Quilizumab, Exacerbations, FEV_1_

## Abstract

**Background:**

Quilizumab, a humanized IgG1 monoclonal antibody, targets the M1-prime segment of membrane-expressed IgE, leading to depletion of IgE-switched and memory B cells. In patients with mild asthma, quilizumab reduced serum IgE and attenuated the early and late asthmatic reaction following whole lung allergen challenge. This study evaluated the efficacy and safety of quilizumab in adults with allergic asthma, inadequately controlled despite high-dose inhaled corticosteroids (ICS) and a second controller.

**Methods:**

Five hundred seventy-eight patients were randomized to monthly or quarterly dosing regimens of subcutaneous quilizumab or placebo for 36 weeks, with a 48-week safety follow-up. Quilizumab was evaluated for effects on the rate of asthma exacerbations, lung function, patient symptoms, serum IgE, and pharmacokinetics. Exploratory analyses were conducted on biomarker subgroups (periostin, blood eosinophils, serum IgE, and exhaled nitric oxide).

**Results:**

Quilizumab was well tolerated and reduced serum total and allergen-specific IgE by 30–40 %, but had no impact on asthma exacerbations, lung function, or patient-reported symptom measures. At Week 36, the 300 mg monthly quilizumab group showed a 19.6 % reduction (*p =* 0.38) in the asthma exacerbation rate relative to placebo, but this was neither statistically nor clinically significant. Biomarker subgroups did not reveal meaningful efficacy benefits following quilizumab treatment.

**Conclusions:**

Quilizumab had an acceptable safety profile and reduced serum IgE. However, targeting the IgE pathway via depletion of IgE-switched and memory B cells was not sufficient for a clinically meaningful benefit for adults with allergic asthma uncontrolled by standard therapy.

**Trial registration:**

ClinicalTrials.gov NCT01582503

**Electronic supplementary material:**

The online version of this article (doi:10.1186/s12931-016-0347-2) contains supplementary material, which is available to authorized users.

## Background

Asthma, a chronic inflammatory disorder of the airways, affects over 300 million people worldwide [1]. Some patients have persistent symptoms despite the use of steroids and other therapies. These patients are at the highest risk for future exacerbations and unscheduled use of healthcare resources [2].

Asthma is a heterogeneous disorder with distinct endotypes, the pathogenic cellular and molecular mechanisms that drive disease in different patient subgroups [3–6]. Allergic asthma is a hypersensitivity driven by the complex interaction of epithelial, dendritic, and type 2 innate lymphoid cells, leading to activation of CD4^+^ T helper 2 (Th2) cells in response to allergen exposure [7]. Th2 cells produce interleukin (IL)-4, IL-5, IL-13, and other cytokines that promote immunoglobulin E (IgE) class switching of B cells, increased IgE synthesis by plasma B cells, and recruitment and activation of eosinophils and other inflammatory cells. When allergen cross-links IgE bound to IgE receptors on mast cells and basophils, cell activation and allergic mediator release occur, which may lead to mucus production and airway hyperreactivity [8]. Biomarkers in sputum, bronchoalveolar lavage, blood, and bronchial biopsies may indicate a particular endotype and potentially identify patients with increased responsiveness to endotype-specific treatments [5, 9]. For example, elevated levels of serum periostin, an extracellular matrix protein induced by IL-4 and IL-13 in airway epithelium, identified a subset of asthma patients who responded better to lebrikizumab, an anti-IL-13 monoclonal antibody [10]. Similarly, patients with high levels of exhaled nitric oxide (NO), blood eosinophils, or serum periostin, had fewer asthma exacerbations following treatment with omalizumab, a neutralizing anti-IgE antibody, than patients with low biomarker levels [11]. These data suggest that IgE may drive allergic asthma in a subset of patients.

Quilizumab is a humanized, monoclonal IgG1 that binds to the M1-prime segment present only on membrane IgE [12], but not on soluble IgE in serum [13–15]. Membrane IgE is expressed on IgE-switched B cells, IgE memory B cells, and IgE plasmablasts, but not on IgE plasma cells [16]. In animal studies, anti-M1 prime bound membrane IgE on IgE-switched B cells and plasmablasts and depleted them through apoptosis and antibody-dependent cell-mediated cytotoxicity, leading to fewer IgE plasma cells and less production of soluble IgE [17].

We previously evaluated the safety, tolerability, and activity of quilizumab in Phase Ia (31 healthy volunteers) [18], Phase Ib (24 allergic rhinitis patients) [19] and Phase IIa (15 mild asthma patients) [19] studies. In these studies, quilizumab reduced serum total IgE by approximately 25 %. These decreases were sustained for at least 6 months after the last dose, in contrast to omalizumab, which must be administered every 2–4 weeks to maintain reduced IgE levels [20]. Quilizumab also abrogated the increase in challenge-specific IgE in patients with mild asthma following a whole-lung allergen challenge, and reduced the early and late asthmatic reactions [19]. Quilizumab may reset the IgE repertoire by targeting IgE production and provide a more sustained effect with a lower dose frequency then omalizumab. The primary purpose of this study was to evaluate the efficacy, safety, and pharmacokinetics of quilizumab after 36 weeks of treatment in adults with allergic asthma inadequately controlled despite high-dose inhaled corticosteroids (ICS) and a second controller.

## Methods

### Trial design

The COSTA trial of quilizumab was a Phase II, randomized, double-blind, placebo-controlled study (including recruiting sites from 14 countries: Argentina, Belgium, Bulgaria, Canada, Germany, Hungary, Mexico, New Zealand, Peru, Poland, Romania, Russia, Ukraine, and the United States) that enrolled 578 adults with uncontrolled allergic asthma.

Patients were randomly assigned (1:1:1:1) to one of three dosing regimens of quilizumab or placebo using an interactive web response system. Randomization was stratified based on serum periostin level (<50 ng/mL, ≥50 ng/mL), exacerbation history (number of exacerbations (1, >1) requiring use of systemic corticosteroids in the prior 18 months), IgE level (≤75 IU/mL, 75–200 IU/mL, >200 IU/mL), and country. Patients and study site personnel were blinded to treatment assignments until all follow-up data through Week 84 were collected and verified.

Quilizumab (Genentech, Inc., South San Francisco, CA) was delivered subcutaneously at nine monthly intervals at 300 mg per dose (300 mg M), or at three quarterly intervals and at Week 4 at 150 or 450 mg per dose (150 or 450 mg Q). The treatment period ended 36 weeks after randomization and was followed by a 48-week period to assess the sustained efficacy and safety of quilizumab (see Additional file 1: Figure S1).

#### Ethics, consent, and permissions

Quorum Review based in Seattle, WA was the primary central IRB used by sites in North America, however multiple other site-specific institutional review boards at other global sites approved the protocol and patients gave written, informed consent. The trial was conducted in full conformance with the International Conference on Harmonisation E6 guidelines and the Declaration of Helsinki, or laws and regulations of the country where the research was conducted, whichever afforded greater protection to the individual.

### Participants

#### Inclusion criteria

Adult asthma patients, aged 18–75 years, were required to have: bronchodilator reversibility of either ≥12 % β-agonist reversibility using 4 puffs of a short-acting β − agonist (SABA) or a PC20 FEV_1_ methacholine (provocative concentration of methacholine producing a 20 % fall in FEV_1_ (forced expiratory volume in 1 s)) of 8 mg/mL or less, within the last 2 years; a pre-bronchodilator FEV_1_ at screening of 40–80 % predicted; daily use of ICS (≥400 μg/day total daily dose of fluticasone propionate or equivalent) and a second controller for a minimum of 3 consecutive months; inadequately-controlled asthma as documented by a 5-item Asthma Control Questionnaire (ACQ-5) [21] score ≥1.50 at screening and randomization, despite compliance with asthma controller therapy; at least one positive aeroallergen-specific IgE (≥0.35 kU(A)/L), or a total serum IgE ≥75 IU/mL; and a history of at least one protocol-defined asthma exacerbation in the 18 months prior to screening. Asthma exacerbations were defined as new or increased asthma symptoms (wheezing, cough, chest tightness, shortness of breath, or nighttime awakening due to symptoms) that led to treatment with systemic corticosteroids for at least 3 days or to hospitalization.

#### Exclusion criteria

Patients were excluded from the study if they had an asthma exacerbation requiring systemic steroids in the 30 days prior to screening, or between screening and randomization, a >20 % relative change in FEV_1_ between screening and randomization, any pre-existing active lung disease other than asthma, any infections, elevated IgE levels for reasons other than allergy, or were former or current smokers.

### Outcome measures

The primary efficacy outcome was the annualized rate of protocol-defined asthma exacerbations from baseline to Week 36.

Secondary efficacy outcomes included assessments of lung function using the relative change in pre-bronchodilator FEV_1_ from baseline to Weeks 12 and 36 and the change in asthma symptoms from baseline to Week 36, using total and daytime symptom severity scores derived from a daily patient diary. The diary was also used to determine the proportion of patients who had no nighttime awakenings due to asthma symptoms and the proportion of patients with fewer than 2 days of SABA use per week by Week 36.

Exploratory outcome measures included the change in asthma control from baseline to Week 36, as measured by the ACQ-5. To assess the change in allergy-related quality-of-life measures from baseline to Week 36, the Standardized Asthma Quality of Life Questionnaire (AQLQ(S)) [22] and the Standardized Rhinoconjunctivitis Quality of Life Questionnaire (RQLQ(S)) [23] were administered at patient visits throughout the treatment and follow-up periods. We also evaluated the ability of biomarkers (serum periostin, blood eosinophils, exhaled NO, and serum IgE) to predict benefit from quilizumab.

Safety outcomes were assessed throughout the 36-week treatment and 48-week follow-up periods.

### Assessments

The patient daily diary included two sections in addition to peak flow measurements: 1) a morning section capturing awakenings and rescue medication use at night, and symptoms on awakening; and 2) an evening section capturing daytime symptom severity, rescue medication use, preventive inhaler use, activity impairment during the day, and nasal symptoms. The modified total asthma symptom score (mTASS) was generated from a subset of diary questions adapted from a previous questionnaire [24], scoring nighttime awakenings (0–3), symptoms on awakening (0–1), and daytime symptom severity (0–4), for a total score range of 0–8. For the analyses of symptom scores, rescue medication use, and nighttime awakenings, daily scores were averaged over the previous 7 days prior to the time point of interest. Baseline values were derived from a minimum of 10 days of patient diary entries during the 14 days prior to the first treatment.

### Pharmacokinetics and immunogenicity

Serum levels of quilizumab for pharmacokinetic assessments were measured using a validated enzyme-linked immunosorbent assay (ELISA; Genentech, Inc., South San Francisco, CA). The pharmacokinetic outcomes included: serum concentrations prior to dosing at Weeks 0, 4, 12, 24, and 32; serum concentrations at Week 5 and Week 25; maximum observed serum concentrations (*C*_*max,obs*_); time of maximum observed serum concentration (*T*_*max, obs*_); and terminal elimination half-life (*t*_*1/2*_). Serum anti-therapeutic antibodies (ATAs) were assessed in samples at Weeks 0, 4, 12, 24, 36, 48, 60, and 84 using a validated bridging ELISA (Genentech, Inc., South San Francisco, CA).

### Biomarkers

Samples for biomarker assessments were collected throughout the study. Allergen-specific and total IgE were measured in serum by ImmunoCAP® Specific IgE blood tests (ViraCor-IBT Laboratories, Lee’s Summit, MO). Specific IgE was measured for the following allergens: cat, house dust mite (HDM) Dermatophagoides farinae, HDM Dermatophagoides pteronyssinus, ragweed, aspergillus, timothy grass, bermuda grass, oak, birch, plantain, and orchard grass. The maximum specific IgE was defined as the specific IgE with the highest titer of specific IgEs pre-dose in each patient. Only observed values of IgE levels were analyzed, with no imputation performed for missing IgE data. Peripheral blood eosinophil counts were obtained from standard complete blood counts. Serum periostin was measured by immunoassay using the Roche Elecsys platform (Roche Diagnostics Ltd., Rotkreuz, Switzerland). Fractional exhaled nitric oxide (FeNO) was measured using a hand-held portable device, NIOX MINO® (Niox; Morrisville, NC), according to American Thoracic Society/European Respiratory Society 2009 guidelines [25].

### Statistical methods

All patients received at least one dose of study drug (intention-to-treat, ITT population) and were included in all safety and efficacy analyses. Demographic and baseline characteristics were summarized using descriptive statistics. The primary endpoint, the annualized exacerbation rate, was calculated by the total number of protocol-defined exacerbations observed in the group over the treatment period divided by total patient-weeks at risk (number of weeks from first study drug administration to the earliest of Week 36 or study discontinuation) for the group and multiplied by 52. For patients who discontinued the study prematurely, there was no imputation of additional exacerbations. The rates of asthma exacerbations were compared between study groups using a Poisson regression with overdispersion model, including terms for periostin status (<50, ≥50 ng/mL), number of prior exacerbations (1, >1), and IgE level (<200, ≥200 IU/mL). For biomarker subgroup analyses, unadjusted asthma exacerbation rates were calculated. Corresponding two-sided p-values and 90 % confidence intervals (CI) were reported.

The relative change in pre-bronchodilator FEV_1_ from baseline was calculated as the absolute change in FEV_1_ (volume in liters) from baseline divided by the FEV_1_ at baseline. For secondary and exploratory end points, the means and standard deviations of all values for relative change were calculated according to study group at Weeks 12 and 36. The mean relative changes from baseline were compared between the study groups using the differences between the means for each group, with the associated two-sided 90 % CIs. Missing values were imputed using the last-observation-carried-forward (LOCF) approach, as prespecified in the statistical analysis plan. An analysis of covariance (ANCOVA) model with factors for baseline level, periostin status (<50 ng/mL, ≥50 ng/mL), number of prior exacerbations (1, >1), and IgE level (<200 IU/mL, ≥200 IU/mL) was fit to assess the treatment effect.

#### Sample size determination

We planned to randomize 560 patients to one of three dose regimens of quilizumab or placebo in a 1:1:1:1 ratio (140 patients per group). This sample size provided approximately 84 % power to detect a 50 % reduction in average exacerbation rates due to quilizumab, assuming 0.63 exacerbations per patient in the placebo group over a 36-week treatment period, and a significance level of α = 0.10. This sample size also provided approximately 70 % power to detect a 50 % reduction in average exacerbation rates in the subgroup of periostin-high patients, assuming 0.69 exacerbations per patient in the placebo group over the 36-week period, a significance level of 0.15, and 50 % of patients in each treatment arm to be periostin high.

## Results

### Patient demographics and study flow

**Fig. 1:**
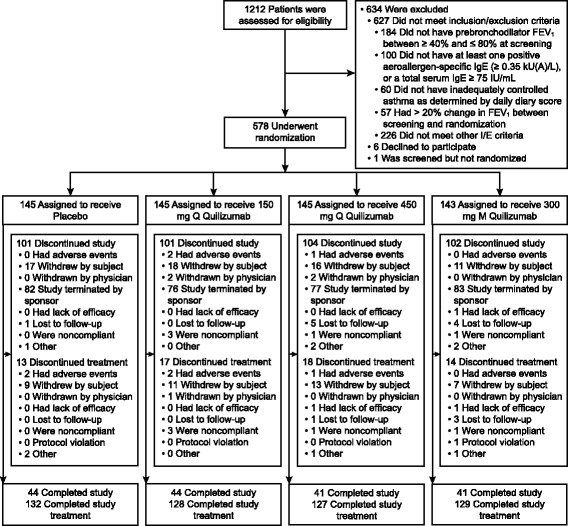
Study flow chart. I/E, inclusion/exclusion; M, monthly; Q, quarterly

1212 patients were screened for eligibility and 578 adult asthma patients (18–75 years old) were enrolled (Fig. 1) from April 2012 to September 2013; the study was completed in November 2014. There were no major imbalances between treatment groups, but the 300 mg monthly quilizumab group had a lower mean age (45 versus 47–48 years) and a lower median IgE level (190 versus 234–254 IU/mL) compared to the other treatment arms (Table 1). 89 % (384/433) of patients who received quilizumab and 91 % (132/145) of patients who received placebo completed the study treatment. The sponsor terminated the trial early because of the lack of efficacy for the primary end-point (asthma exacerbations) at Week 36, with the median time in the study at 72 weeks.

**Table 1:** Patient demographics/baseline characteristics

	Placebo (*n* = 145)	Quilizumab 150 mg Q (*n* = 145)	Quilizumab 450 mg Q (*n* = 145)	Quilizumab 300 mg M (*n* = 143)	All Patients (*N* = 578)
Age (y)	47.9 (13.0)	46.8 (13.3)	47.0 (13.6)	44.8^a^ (12.2)	46.6 (13.1)
Gender (Female)	84 (58 %)	89 (61 %)	98 (68 %)	86 (60 %)	357 (62 %)
Race (White)	122 (84 %)	119 (82 %)	117 (81 %)	114 (80 %)	472 (82 %)
Weight (kg)	82.8 (23.3)	81.4 (18.13)	80.3 (21.4)	81.3 (19.4)	81.4 (20.6)
Previous number of exacerbations					
1	90 (62 %)	88 (61 %)	91 (63 %)	89 (62 %)	358 (62 %)
2	30 (21 %)	33 (23 %)	31 (21 %)	28 (20 %)	122 (21 %)
≥3	25 (17 %)	24 (17 %)	23 (16 %)	26 (18 %)	98 (17 %)
FEV_1_ % predicted	60.8 (11.9)	58.6 (12.2)	61.2 (12.8)	63 (12.7)	60.9 (12.5)
FEV_1_ % reversibility	19.6 (15.8)	23.3 (20.6)	21.6 (18.8)	23.4 (18.0)	22 (18.4)
IgE (IU/mL) median	234.0	254.0	249.5	190.0^a^	232.5
Periostin (ng/mL) median	51.0	53.4	52.4	52.1	51.9
FeNO (ppb)	33.4 (32.0)	35.7 (36.26)	33.8 (32.1)	35.2 (32.5)	34.5 (33.2)
Eosinophils (cells/μl) median	210	220	220	240	220

Mean (SD) unless otherwise noted

^a^The 300 mg M treatment group had a lower age and IgE level

*M* monthly, *Q* quarterly

### Safety

Quilizumab was well tolerated, and the number of patients experiencing at least one adverse event (AE) was comparable between all treatment groups (Table 2). Through Week 36, the most frequent AEs across all quilizumab dose groups were worsening of asthma (27 %), nasopharyngitis (15 %), upper respiratory tract infection (7 %), bronchitis (5 %), sinusitis (5 %), and injection site pain (5 %).

**Table 2:** Safety, from first dose of study drug through end of study (Week 84)

	Placebo (*n* = 145)	Quilizumab 150 mg Q (*n* = 145)	Quilizumab 450 mg Q (*n* = 145)	Quilizumab 300 mg M (*n* = 143)	All Quilizumab (*n* = 433)
Any AE	114 (78.6 %)	110 (75.9 %)	108 (74.5 %)	103 (72.0 %)	321 (74.1 %)
Serious AE	12 (8.3 %)	11 (7.6 %)	10 (6.9 %)	16 (11.2 %)	37 (8.5 %)
Grade 3 AE or higher	21 (14.5 %)	28 (19.3 %)	30 (20.7 %)	24 (16.8 %)	82 (18.9 %)
Grade 2 AE or higher	86 (59.3 %)	78 (53.8 %)	80 (55.2 %)	82 (57.3 %)	240 (55.4 %)
Infections and infestations	76 (52.4 %)	73 (50.3 %)	71 (49.0 %)	68 (47.6 %)	212 (49.0 %)
Parasitic infections	0	0	0	1 (0.7 %)	1 (0.2 %)
Serious infections	3 (2.1 %)	3 (2.1 %)	1 (0.7 %)	2 (1.4 %)	6 (1.4 %)
Injection site reactions	12 (8.3 %)	10 (6.9 %)	13 (9.0 %)	7 (4.9 %)	30 (6.9 %)
Serious injection site reactions	0	0	0	0	0
AE leading to treatment withdrawal	1 (0.7 %)	4 (2.8 %)	1 (0.7 %)	1 (0.7 %)	6 (1.4 %)
Malignancies	0	2 (1.4 %)	2 (1.4 %)	0	4 (0.9 %)
Deaths	0	0	0	0	0

*AE* adverse event, *M* monthly, *Q* quarterly

The treatment-emergent ATA rate was 1.4 % in both the 150 mg Q and 300 mg M groups, and 2.1 % in the 450 mg Q group. ATA incidence for all dosing groups was 1.6 % (7 of 427 quilizumab-dosed patients). There was no apparent impact of positive ATA results on either pharmacokinetic profiles or safety.

### Pharmacokinetics

Quilizumab displayed linear pharmacokinetics with a dose-proportional increase in exposure as shown by increases in *C*_*max*_ values between the 150 and 450 mg Q doses at Weeks 5 and 25. (see Additional file 1: Figure S2). At treatment Weeks 4, 12, and 24, the mean trough serum concentrations of quilizumab were: (150 mg Q) 5.7 ± 2.3 μg/mL, 2.6 ± 2.0 μg/mL, and 1.3 ± 4.6 μg/mL, respectively; (450 mg Q) 17.1 ± 7.0 μg/mL, 7.2 ± 3.8 μg/mL, and 2.3 ± 2.2 μg/mL, respectively; and (300 mg M) 11.6 ± 4.5 μg/mL, 16.7 ± 6.7 μg/mL, and 16.8 ± 7.7 μg/mL, respectively. For the 150 mg Q, 450 mg Q, and 300 mg M doses, the mean *C*_*max,obs*_ values were 18 ± 7.8 μg/mL, 51.6 ± 19.5 μg/mL, and 34 ± 12.6 μg/mL, which occurred at *T*_*max,obs*_ of 36.4 ± 4.3 days, 36.2 ± 3.0 days, and 36.2 ± 3.5 days, respectively, approximately 7 days following the second doses. Based on non-compartmental analysis, the mean *t*_*1/2*_ values of quilizumab across the dose groups ranged from 16.4 to 18.7 days, which suggests that steady-state was attained by the time the final doses were administered for both monthly and quarterly regimens.

### Pharmacodynamic biomarkers

**Fig. 2:**
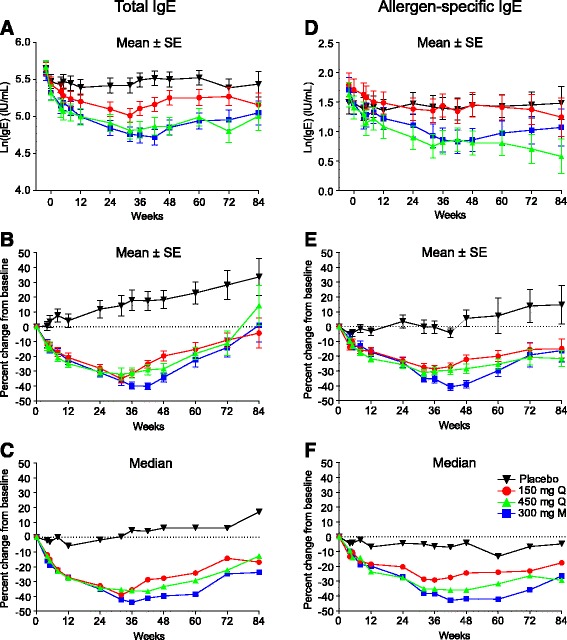
Effect of quilizumab on serum total (**a**-**c**) and allergen-specific IgE (**d**-**f**) from baseline to Week 84. IgE levels were represented as the mean ± standard error (SE) of the natural logarithm (**a**, **d**), the mean ± SE percent change from baseline (**b**, **e**), and median percent change from baseline (**c**, **f**). All IgE data analysis was based on observed values. M, monthly; Q, quarterly

Quilizumab gradually reduced mean serum total and allergen-specific IgE, reaching a 30–40 % reduction at Week 36 in all three quilizumab cohorts (*p* < 0.01 compared to placebo; Fig. 2). The largest decreases in total and allergen-specific IgE were observed at Week 42 in the 300 mg M treatment cohort. Serum total and allergen-specific IgE in quilizumab-treated patients gradually increased throughout the safety follow-up period, but did not return to pre-dose levels by the end of the study period (Week 84).

Peripheral blood eosinophils and FeNO levels were not modified following quilizumab treatment (see Additional file 1: Figure S3).

### Asthma exacerbation rates

**Fig. 3:**
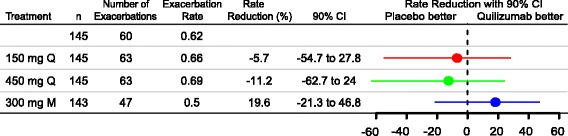
Rate of protocol-defined asthma exacerbations through Week 36 for all patients. M, monthly; Q, quarterly

Quilizumab treatment did not produce a clinically meaningful reduction of the rate of asthma exacerbations over the 36-week treatment period. In the ITT population, the reduction in the asthma exacerbation rate relative to placebo was 19.6 % (90 % CI: −21 to 47; *p* = 0.38), −11.2 % (90 % CI: −63 to 24; *p* = 0.65), and −5.7 % (90%CI: −55 to 28; *p* = 0.81) in the 300 mg M, 450 mg Q, and 150 mg Q quilizumab treatment arms, respectively (Fig. 3).

**Fig. 4:**
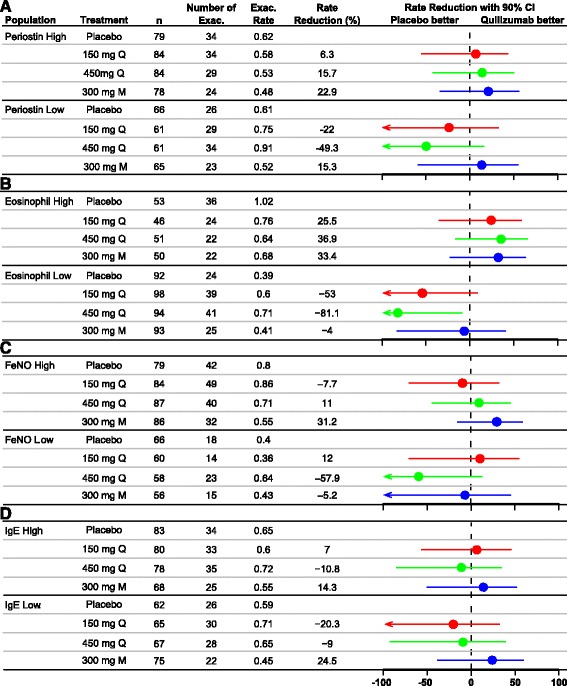
Rate of protocol-defined asthma exacerbations through Week 36 for patients stratified by biomarkers (**a**: periostin; **b**: blood eosinophils; **c**: FeNO; **d**: serum IgE). Exac, exacerbation; M, monthly; Q, quarterly

Subgroup analysis by baseline serum periostin (≥50 ng/ml, <50 ng/ml), blood eosinophils (≥300 cells/μl, <300 cells/μl), FeNO (≥20 ppb, <20 ppb) and serum total IgE (≥200 IU/mL, <200 IU/mL) demonstrated no consistent effect of quilizumab on the exacerbation rate across all doses when compared to the ITT population (Fig. 4).

### Lung function

**Fig. 5:**
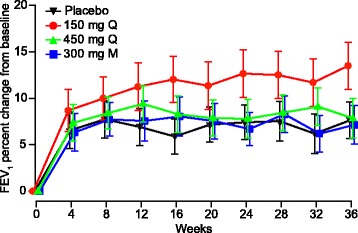
Mean percent change in pre-bronchodilator FEV_1_ from baseline. FEV_1_ for each treatment arm was measured through Week 36. Error bars represent the standard error of the mean. M, monthly; Q, quarterly

No significant evidence of improved FEV_1_ at Weeks 12 and 36 was observed in quilizumab-treated patients compared to placebo (Fig. 5). The highest relative improvement compared to placebo occurred in the lowest dose arm (150 mg Q), but no meaningful improvements were observed in the higher dose arms (450 mg Q and 300 mg M). Relative changes from baseline in FEV_1_ compared to placebo were 3.8 % (90 % CI: −1.2 to 8.9, *p* = 0.21) at Week 12 and 5.6 % (90 % CI: 0.6 to 10.6, *p* = 0.07) at Week 36 in the 150 mg Q treatment arm; 2.2 % (90 % CI: −2.8 to 7.2, *p* = 0.48) at Week 12 and 0.1 % (90 % CI: −5.0 to 5.1, *p* = 0.98) at Week 36 in the 450 mg Q arm; and 1.1 % (90 % CI: −3.9 to 6.1, *p* = 0.72) at Week 12 and 0.1 % (90 % CI: −4.9 to 5.2, *p* = 0.96) at Week 36 in the 300 mg M arm (Fig. 5; see Additional file 2: Table S1 and Additional file 3: Table S2).

Analysis of FEV_1_ in the biomarker subgroups demonstrated no consistent or meaningful improvements of FEV_1_ in the quilizumab-treated arms over placebo (see Additional file 2: Table S1 and Additional file 3: Table S2).

### Patient-reported outcomes

During the 36-week treatment period, no meaningful differences were observed between the quilizumab dose groups compared with the placebo group for the total mTASS, daytime mTASS, ACQ-5, AQLQ(S), or RQLQ(S) scores (Table 3).

**Table 3:** Change over 36 weeks in patient-reported outcomes (ITT population)

		Quilizumab placebo-corrected changes		
Endpoint	Placebo (*n* = 145)	150 mg Q (*n* = 145)	450 mg Q (*n* = 145)	300 mg M (*n* = 143)
Mean change in total asthma symptoms score (mTASS)	−1.5	0.1, *p* = 0.58	0.1, *p* = 0.55	−0.1, *p* = 0.51
Mean change in daytime asthma symptoms score (mTASS)	−0.7	0.1, *p* = 0.55	0, *p* = 0.70	0, *p* = 0.69
Mean change in ACQ-5 score	−1.2	0, *p* = 0.86	0, *p* = 0.83	−0.1, *p* = 0.52
Mean change in AQLQ(S) score	1.1	−0.1, *p* = 0.67	0.1, *p* = 0.73	0.3, *p* = 0.05
Mean change in RQLQ(S) score	−0.6	0, *p* = 0.96	−0.1, *p* = 0.35	−0.3, *p* = 0.04
Proportion of patients at Week 36 with no nighttime awakenings in the previous week	0.43	−0.07, *p* = 0.21	−0.02, *p* = 0.69	0, *p* = 0.97
Proportion of patients at Week 36 with ≤2 days of rescue medication use in the previous week	0.42	0.01, *p* = 0.88	0.01, *p* = 0.86	0.06, *p* = 0.33

*ITT* intention-to-treat, *M* monthly, *Q* quarterly

Treatment with quilizumab failed to increase either the proportion of patients who used a SABA for fewer than 2 days in the prior week or the proportion of patients who did not awaken at night due to asthma symptoms for all dosing levels relative to the placebo group at Week 36 (Table 3).

## Discussion

In this study, we evaluated the efficacy and safety of quilizumab, a monoclonal antibody that targets the M1-prime segment of membrane IgE, in adult patients with allergic asthma that was inadequately controlled with standard therapy. After 36 weeks of treatment, quilizumab was well tolerated, with a safety profile consistent with previous clinical studies [18, 19].

Quilizumab demonstrated clear pharmacological activity by reducing both serum total and allergen-specific IgE an average of 30–40 % from baseline in all three treatment arms, in agreement with our previous work [18, 19]. However, quilizumab treatment did not show consistent and clinically meaningful benefits in reducing asthma exacerbations or improving lung function, symptoms, or quality of life. Subgroup analysis in patients with elevated serum periostin, blood eosinophils, FeNO, or serum IgE did not demonstrate a consistent treatment effect for quilizumab.

It is important to understand that reducing serum IgE levels is not quilizumab’s proposed mechanism of action. Quilizumab did not reduce serum IgE as effectively as omalizumab (89–98 % reduction of median free IgE) [26], but these two antibodies target the IgE pathway in distinctly different ways. Quilizumab targets both B-cell switching to IgE and the new stimulation of IgE memory B cells and plasmablasts that lead to enhanced IgE production, processes that occur locally in the airways of allergic patients [16]. Quilizumab’s ability to suppress the challenge-specific increase in serum IgE in our whole lung allergen challenge study in mild asthmatics supports this mechanism [19]. We therefore hypothesize that quilizumab prevents the formation of short-lived IgE plasma cells in the airway. Long-lived IgE plasma cells, which lack IgE on the surface, are not targeted by quilizumab and may be responsible for the remaining serum IgE detected in our studies [16]. Another source of IgE not eliminated by quilizumab may be a subpopulation of IgG1 memory B cells that undergo a secondary switch to IgE when reactivated, and thereby become IgE memory cells [27].

The current study assessed the relevance of this mechanism on exacerbations in patients with inadequately controlled allergic asthma. In the EXTRA study that examined a similar patient population, omalizumab significantly reduced asthma exacerbations by 25 % in the all-patients group [24], by 53 % in the FeNO-high subgroup, and by 32 % in the eosinophil-high subgroup, relative to placebo. In addition, asthma exacerbations were substantially reduced in the periostin-high subgroup (30 %) relative to placebo [11]. In contrast, in our study, quilizumab did not show significant clinical benefit in this population (Fig. 3) or in any subgroup (Fig. 4), possibly because local IgE production by short-lived IgE plasma cells is not critical for exacerbations and other clinical endpoints. For this to be true, we must assume that: 1) quilizumab depleted all M1-prime-positive B cells, and 2) the doses in this study were sufficient to deplete all M1-prime-positive cells. Despite our extensive efforts, we have not been able to accurately detect M1-prime-positive cells in patients with asthma [19], so we have no direct evidence of depletion. However, all three quilizumab dose regimens led to a similar reduction in serum IgE levels, suggesting that quilizumab depleted all target cells, and indicating that higher doses would not have provided additional activity. Interestingly, after stopping quilizumab dosing, serum IgE levels gradually increased, indicating that IgE-switching and formation of short-lived IgE plasma cells may occur continuously in this patient population, a finding not previously reported.

The allergen-challenge model used in our previous studies where quilizumab was shown to be effective [19] may not represent the asthma processes occurring in the patient population in this study, especially given that alternate sources of IgE appear to play a more dominant role. However, the model was key in first demonstrating the effectiveness of omalizumab in patients with mild asthma, where omalizumab significantly reduced both the early and late asthmatic responses following allergen challenge and reduced serum IgE almost completely [28, 29]. Although less effective than omalizumab in a similar population, quilizumab showed reductions of 26 and 36 % in the early and late asthmatic responses, respectively, and a 25 % reduction in serum IgE [19]. Allergen-challenge studies are generally exploratory and are designed to follow biomarkers that trend with early and late asthmatic responses. They are usually conducted in patients with mild asthma, which is not the typical target population for new biologic therapies. At least one therapy, mepolizumab (anti-IL-5), is effective for reducing asthma exacerbations in patients with eosinophilic asthma, yet does not appear to affect early/late asthmatic responses during allergen challenge [30, 31]. It is therefore crucial that appropriate patient populations and outcome measures are selected for future trials.

Whether quilizumab could have a clinically meaningful effect in another subgroup of allergic asthma patients or whether a treatment period beyond 36 weeks would show efficacy, are questions we are unable to address with the current study. The type 2 biomarkers, serum periostin, blood eosinophils, and FeNO, which identified patients with increased clinical benefit from lebrikizumab and omalizumab [10, 11], did not consistently enrich for increased benefit from quilizumab in this study. Similarly, patients with elevated serum IgE levels at baseline did not respond better to quilizumab nor was there a correlation between serum IgE reduction and exacerbation reduction or FEV_1_ improvement (data not shown).

## Conclusions

In adults with uncontrolled allergic asthma, a 36-week treatment with quilizumab, an anti-M1-prime monoclonal antibody that targets IgE-switched and IgE memory B cells, did not result in a clinically significant impact on exacerbation rate, lung function, or quality of life. Our data on quilizumab activity indicate that there are major pathological mechanisms that extend beyond the new, local production of IgE in patients with inadequately controlled allergic asthma.

## Additional files

Additional file 1:
**Figure S1.** Study design. **Figure S2.** Pharmacokinetics of quilizumab in asthma patients. **Figure S3.** Effect of quilizumab on blood eosinophils (A, B) and FeNO (C, D) from baseline to Week 36. (PDF 422 kb) Additional file 2: Table S1. FEV_1_ at Week 12 (all patients and biomarker subgroups). (XLSX 12 kb) Additional file 3: Table S2. FEV_1_ at Week 36 (all patients and biomarker subgroups). (XLSX 48 kb)

## Abbreviations

ACQ-5: 5-item asthma control questionnaire
AE: adverse event
ANCOVA: analysis of covariance
AQLQ(S): standardized asthma quality-of-life questionnaire
ATA: anti-therapeutic antibody
*C*_*max:obs*_: maximum observed serum concentration
ELISA: enzyme-linked immunosorbent assay
FeNO: fractional exhaled nitric oxide
FEV_1_: forced expiratory volume in 1 s
HDM: house dust mite
ICS: inhaled corticosteroid
Ig: immunoglobulin
IL: interleukin
ITT: intention-to-treat
LOCF: last observation carried forward
LSM: least squares mean
mTASS: modified total asthma symptom score
RQLQ(S): standardized rhinitis quality-of-life questionnaire
SABA: short-acting β-agonist
*t*_*1/2*_: terminal elimination half-life
Th2: T helper 2
*T*_*max*:*obs*_: time of maximum observed serum concentration
