# Supplementary material for: A randomized trial of the efficacy and safety of quilizumab in adults with inadequately controlled allergic asthma
Source: Respir Res. 2016 Mar 18;17:29. doi: 10.1186/s12931-016-0347-2 (PMC4797126; doi:10.1186/s12931-016-0347-2)
Supplement: Additional file 1: — Figure S1. Study design. Figure S2. Pharmacokinetics of quilizumab in asthma patients. Figure S3. Effect of quilizumab on blood eosinophils (A, B) and FeNO (C, D) from baseline to Week 36. (PDF 422 kb) [file 12931_2016_347_MOESM1_ESM.pdf]

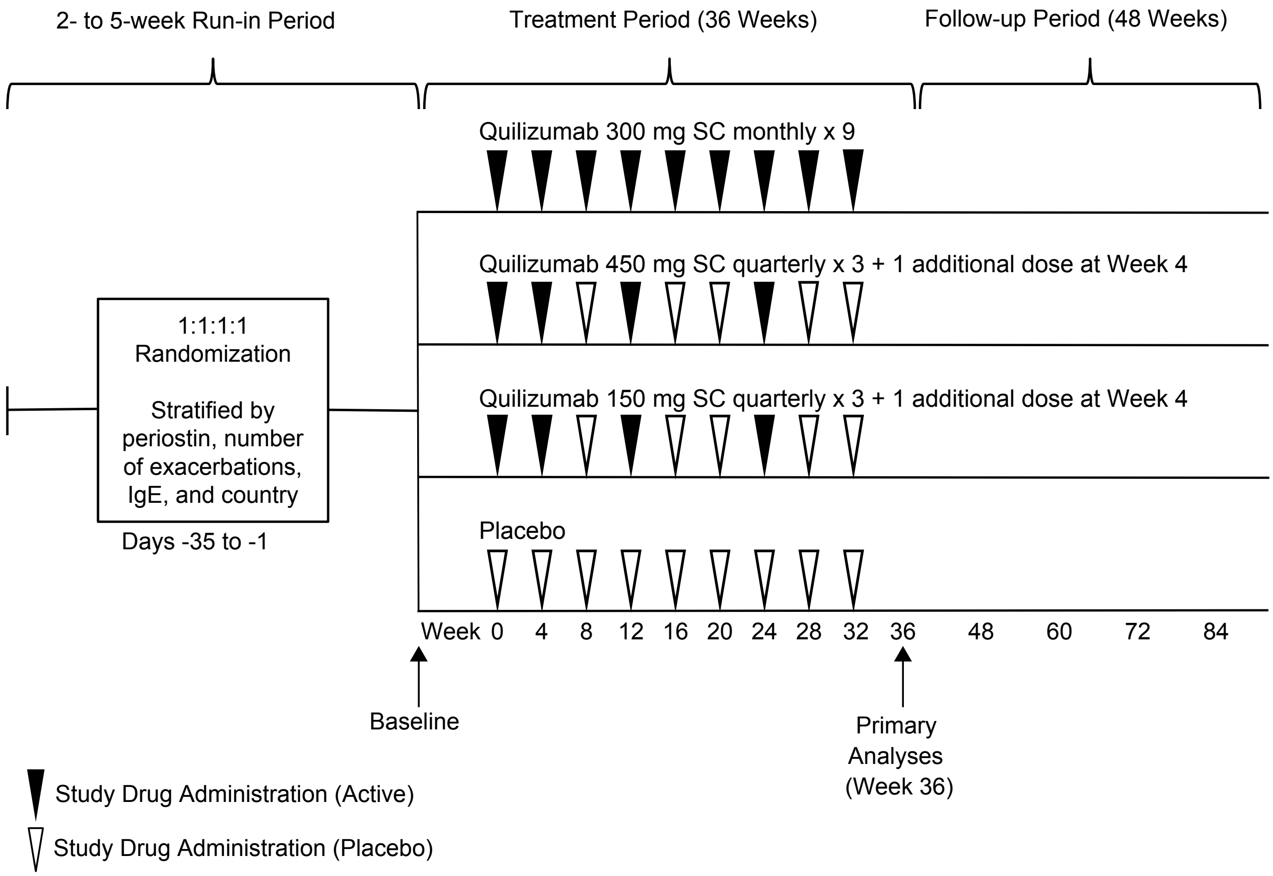

Figure S1. Study design. SC, subcutaneous.

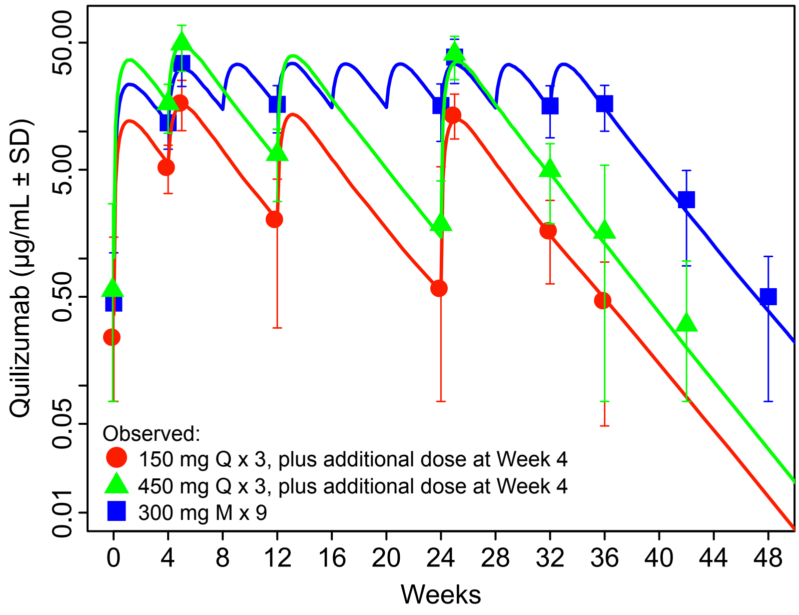

Figure S2. Pharmacokinetics of quilizumab in asthma patients. Symbols indicate observed values; lines indicate predicted values. M, monthly; Q, quarterly; SD, standard deviation.

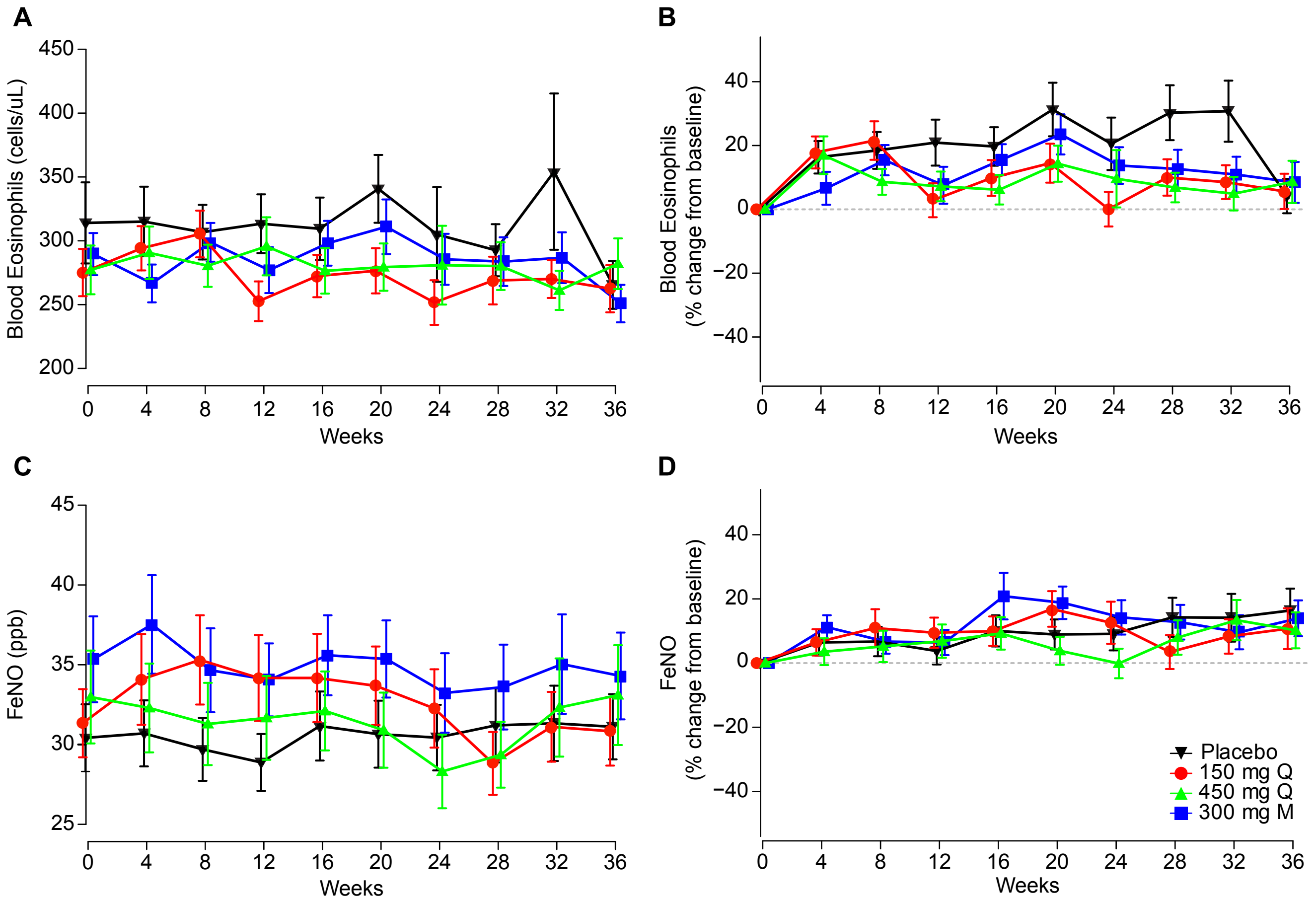

Figure S3. Effect of quilizumab on blood eosinophils (A, B) and FeNO (C, D) from baseline to Week 36. Levels were represented as the mean ± standard error (SE; A, C) and the mean ± SE percent change from baseline (B, D). All data analysis was based on observed values. M, monthly; Q, quarterly.
